# Interruption of Heart’s Action

**Published:** 1860-03

**Authors:** B. Woodward

**Affiliations:** Galesburg


### INTERRUPTION OF HEART'S ACTION. CAUSE—MASTURBATION.

By B. WOODWARD, M. D., Galesburg.

On the evening of 22d Dec. last, I was sent for to see ——, age 2[illegible]. For several weeks past he had been in poor health; very pale and feeble. Found him in bed complaining of great weakness. No pain in any part; tongue clean and moist, but verypallid; conjunctiva and pro-labia blanched; countenance anxious. His pulse was but 32 per minute, and very labored; respiration 8 per minute. Auscultation revealed no organic disease, either of lungs or heart. As no apparent cause could be found for this depressed condition, I questioned him as to his habits, when he acknowledged the practice of masturbation, and that it was from the last act, twenty-four hours previously, that he had been aware of a sense of sinking.

| | | |
|---|---|---|
| ℞ | Brandy, | ℥ iv. |
| | Spts. Ammon. Arom. | ℥ i. |
| | Quinia, | ℈j. |

A teaspoonful to be taken every half hour.

His pulse continued to sink till 11 P. M., when it was but 28 per minute, and respirations 5, with rapid failure of every vital power. Ordered Quinia, grs. v., Brandy, ℥ ss., every hour, with sinapisms to the extremities. This treatment continued for 12 hours brought the pulse to 45, respiration to 10, every third, and sometimes every third and fourth beat of the pulse intermitted. ℞, Quinia ℈jss., Iron by Hydrogen, grs. xxiv. in Cht. No. xii. One to be taken every three hours with Brandy ℥ ss., Arom. spt. Ammon. gtt. x, with each powder. This for 48 hours brought the pulse to 65, intermissions as before, respirations 15. Continued treatment 24 hours longer; pulse 70, every fourth beat wanting. Omit Brandy and spts. Ammon., and continue Quin. et ferri. Twenty-four hours later, pulse 75, rather labored, fourth beat occasionally wanting; to take Quinia, grs. iij., Iron by Hydrogen, grs. ij every six hours.

Jan. 1st. He is walking about comfortable, but weak; advised him to omit quinia, and continue the iron for some days. Gave him a good lecture on habits and dismissed the case. Up to this time, Jan. 27th, he has been constantly improving, and thinks he is cured of his bad propensity. Though I have been familiar with the victims of this vice, and watched its effects, I have never before witnessed such a sudden depression, or derangement of the heart's action; neither do I fully understand it. The prostrated anæmic condition, *gradually* obtained, I can understand, but not the *sudden* failure.

In his case there was no derangement in the functions of the brain, the memory was perfect, and to within forty-eight hours of my being called he had continued his occupation, which required constant thought and attention.

